# The role of minimally invasive surgery in gynaecological cancer: an overview of current trends

**DOI:** 10.52054/FVVO.16.1.005

**Published:** 2024-03-28

**Authors:** D Balafoutas, N Vlahos

**Affiliations:** 2nd Department of Obstetrics and Gynecology, National and Kapodistrian University of Athens, Vasilissis Sofias 76, 115 28, Athens, Greece

**Keywords:** Oncology, laparoscopy, ovarian cancer, cervical cancer, endometrial cancer, MIS, laparoscopy, gynaecological, oncology

## Abstract

**Background:**

The capabilities of minimally invasive surgery, either as conventional laparoscopy, or as robotic surgery, have increased to an extent that it enables complex operations in the field of gynaecological oncology.

**Objective:**

To document the role of minimally invasive gynaecological surgery in cancer.

**Materials and Methods:**

A review of the literature that shaped international guidelines and clinical practice.

**Main outcome measures:**

Current guidelines of major international scientific associations and trends in accepted clinical practice.

**Results:**

In recent years, evidence on oncologic outcome has limited the role of minimally invasive techniques in cervical cancer, while the treatment of early endometrial cancer with laparoscopy and robotic surgery has become the international standard. In ovarian cancer, the role of minimally invasive surgery is still limited. Current evidence on perioperative morbidity underlines the necessity to implicate minimally invasive techniques whenever possible.

**Conclusion:**

The optimal surgical route for the treatment of gynaecological cancer remains in many cases controversial. The role of minimally invasive surgery remains increasing in the course of time.

**What is new?:**

This comprehensive review offers an entire perspective on the current role of minimally invasive surgery in gynaecological cancer therapy.

## Introduction

Minimally invasive surgery (MIS) has been widely adopted, and it has become the standard procedure in many benign gynaecologic conditions. Its role in the management of gynaecologic malignancies has been explored since the beginning of operative laparoscopy (Mangioni et al., 1979) and has been expanding ever since (Conrad et al., 2015). For early-stage endometrial cancer, the laparoscopic approach is already the gold standard and is recommended by most society guidelines (Amant et al., 2018; Concin et al., 2021). The role of MIS and, in the recent years of robotic surgery, in the management of cervical and even ovarian cancer is currently under evaluation, with the laparoscopic evaluation prior to resection of ovarian neoplasms being widely accepted (Armstrong et al., 2021).

The advantages of MIS over open surgery in the fields of postoperative morbidity and quality of life are no longer disputed (Kotani et al., 2021). Additionally, modern perioperative management protocols favouring prompt postoperative mobilization and hospital discharge after MIS (Kim et al., 2022) can reduce the negative effect of the surgical stress and shorten the delay to adjuvant chemotherapy (St-Amour et al., 2020).

The gynaecological tumours constitute a heterogenous group regarding biology, risk factors, prognosis, and management, however they share neighbouring anatomical regions and, most importantly, are treated by the same surgeons. Therefore, it is interesting to offer an overview of the role and limitations of MIS in diagnostic and therapeutic procedures for gynaecological cancer.

Regarding the search methodology for this narrative review, we focused on the scientific publications and resources that shaped the current trends in the management of various gynaecological malignancies. The references of major international guidelines, committee opinions and practice bulletins were screened in the fields of cervical, endometrial, and ovarian cancer. Guidelines from the American College of Obstetricians and Gynecologists (ACOG), the Society of Gynecologic Oncology (SGO), the European Society for Medical Oncology (ESMO), the European Society of Gynaecological Oncology (ESGO), the British Gynaecological Cancer Society (BGCS) and the German Society for Obstetrics and Gynecology (DGGG) were analysed focusing on the possible applications of MIS in the above fields. Relevant resources were selected by the authors and completed with additional recent publications based on relevance and importance. The conclusions are categorized depending on the localization of the tumour.

## Cervical cancer

Cervical cancer is the third most common gynaecologic malignancy (Ferlay et al., 2019) and one of the leading cases of cancer-related death among young women. The surgical treatment for early stages has been described by Ernst Wertheim more than 100 years ago (Dursun et al., 2011). The importance of wide local excision of tissues around the tumour was recognized early and the modification of the procedure by Joe Vincent Meigs with the extensive pelvic lymph node dissection did improve the surgical outcome. However, this operation is associated with significant morbidity, including blood loss, thromboembolism and prolonged hospital stay. In the recent years with the overall improvement of MIS, these techniques have been proposed to reduce the perioperative morbidity (Basaran et al., 2015). The first laparoscopic radical hysterectomy was described by Nezhat et al. (1992) followed by reports on the safety and favourable oncologic outcome of the procedure by others (Sedlacek et al., 1994; Spirtos et al., 2002; Ting, 1994).

For patients with stage IA1 cervical cancer and no evidence of lymphovascular space invasion (LVSI) after a conization procedure the treatment of choice is a simple hysterectomy. This can be performed per laparotomy, however vaginal, laparoscopic, and robotic-assisted approaches represent viable alternatives. This group of patients is at low risk for lymph node involvement and pelvic lymphadenectomy is not typically indicated (Marth et al., 2017). Stage IA1 patients with LVSI and IA2 patients should be treated with modified radical hysterectomy (Querleu-Morrow Classification Type B) with lymphadenectomy (Querleu et al., 2017; Querleu and Morrow, 2008), whereas simple hysterectomy may be an acceptable alternative for some patients. Some centres have replaced lymphadenectomy with sentinel lymph node biopsy (SLNB) and due to the feasibility of SLNB with laparoscopy, some surgeons will choose this route, in particular because of the very high detection rates with the indocyanine green (ICG) method (Kim et al., 2018). The ovaries can be preserved in young patients with squamous cell tumours and should be removed in those with adenocarcinoma. Laparoscopy offers an adequate intraoperative ovarian evaluation in case of ovarian preservation and is best suited for the additional procedure if indicated.

Patients with stage IB1 and IB2 cervical cancers will typically undergo a radical hysterectomy (Querleu-Morrow Classification Type C). Most centres will currently offer a laparotomy for this procedure. This resurgence of open surgery is based on the Laparoscopic Approach to Cervical Cancer (LACC) trial, a large, randomized trial with 631 patients with stage IA1 (with LVSI), IA2, and IB1 cervical cancer who were assigned to radical hysterectomy using MIS or laparotomy (Ramirez et al., 2018). Patients in the MIS compared with laparotomy group had similar rates of postoperative adjuvant therapy. The MIS group had a lower disease-free survival (DFS) at 4.5 years (86 versus 96.5 percent) and lower DFS (91.2 versus 97.1 percent), overall survival (93.8 versus 99 percent), and a higher rate of death from cervical cancer (4.4 versus 0.6 percent) at three years. Almost 40% of all the recurrences in both study groups were in the vaginal vault or the pelvis, and all the non- vaginal vault pelvic recurrences were in the MIS arm. Inferior DFS was observed in both subgroups of the MIS surgery arm that had robot-assisted (n = 45) or laparoscopic (n = 244) radical hysterectomy. In summary, the patients randomized to MIS had a nearly fourfold increase in the risk of recurrence (hazard ratio [HR], 3.7; 95% CI, 1.6–8.6) and a sixfold increase in the risk of death (HR, 6.0; 95% CI, 1.77–20.3).

Interestingly, in the same issue of The New England Journal of Medicine, an observational retrospective Study was published based on national databases of the United States (Melamed et al., 2018). This study demonstrated a higher mortality rate among women undergoing MIS compared with open radical hysterectomy (9.1% vs 5.3% mortality risk in 4 years; HR, 1.65; 95% CI, 1.22–2.22). These results were succeeded by several retrospective analyses (Kim et al., 2019; Paik et al., 2019; Uppal et al., 2020) reporting inferior oncologic outcomes for MIS. The controversy over the possible advantages of MIS for cervical cancer was also fuelled by an additional study, which described no difference in the quality of life 6 weeks and 3 months after surgery in the patient collective of the LACC Trial (Frumovitz et al., 2020). A later, large meta-analysis included 49 studies and 2675 patients and showed that the hazard of recurrence or death was 71% higher among patients who underwent minimally invasive radical hysterectomy compared with those who underwent open surgery (hazard ratio [HR], 1.71; 95% CI, 1.36-2.15; p < .001), and the hazard of death was 56% higher (HR, 1.56; 95% CI, 1.16- 2.11; p = .004) (Nitecki et al., 2020). These results also supported the LACC Trial conclusions regarding IA1 to IIA disease. In the European SUCCOR trial, minimally invasive radical hysterectomy had double the risk of recurrence (HR, 2.07; 95%CI, 1.35 to 3.15; p = 0.001) and death (HR, 2.45; 95%CI, 1.30 to 4.60, p = 0.005) compared to abdominal radical hysterectomy for stage IB1 cervical cancer (Chiva et al., 2020).

The above results caused a shift of the treatment of early-stage cervical cancer back to laparotomy: Despite ongoing substantial controversy, the rates of MIS radical hysterectomy fell dramatically and pervasively after the LACC trial presentation, from 51.9% to 27.1% (RR 0.52, 95% CI 0.47, 0.58; p < 0.0001) (Charo et al., 2020). At the same time, many scientific societies reacted to the presented data with adjusting their guidelines on cervical cancer surgical therapy: ESGO (Querleu et al., 2020), FIGO (2020) and NCCN (Koh et al., 2019) stated that open abdominal radical hysterectomy should be the current the standard of care for the treatment of early stage cervical cancer.

Despite this development, there is some concern that the exclusion of MIS for the treatment of smaller early-stage cervical cancer is not fully justified. The authors of the LACC trial report noted that the trial was underpowered to evaluate outcomes for tumour size <2 cm. Indeed, only 8% of the LACC-collective patients had Stage IA1/ IA2 disease (51 patients) and in a sub-analysis by tumour size and treatment group in patients who recurred, there was no statistically significant difference in survival for the Stage IA1/IA2 subset (14% abdominal, 19% MIS group, p = 0.90). Similarly, in a sub-analysis of the SUCCOR trial, patients with tumours <2 cm did not differ in the progression-free survival (PFS) (HR, 1.63; 95% CI 0.79–3.40; p = 0.19) and OS (HR, 2.77; 95% CI, 0.91–8.47; p = 0.072) when comparing minimally invasive versus abdominal radical hysterectomy.

In a recent Canadian retrospective cohort study of patients undergoing hysterectomy (radical and non-radical) for microinvasive cervical cancer (FIGO 2018 stage IAI/IA2), no significant difference in 5-year PFS was found (96.7% MIS, 93.7% abdominal, 90.0% combined vaginal- laparoscopic, p = 0.34) (Piedimonte et al., 2022). In a sub-analysis of patients with IA1 LVSI+/IA2 (n = 186), survival results were similar. A further recent retrospective analysis of data collected before the LACC trial reported no differences for recurrence or death depending on surgical route while demonstrating significant higher blood loss in the laparotomy group (Pecorino et al., 2022).

In conclusion, the hysterectomy route for cervical cancer remains controversial and the conduct of future clinical trials comparing MIS with laparotomy is challenging because of the current evidence and the subsequent difficulty in medical ethics approvals. The employment of robotic surgery is promising in this field. Retrospective analyses including meta-analyses suggest that the oncologic safety of open surgery and robot-assisted laparoscopic surgery are comparable (Shazly et al., 2015). These data and the hypothesis that the use of intra-uterine manipulators and intra-corporeal colpotomy may account for the negative outcomes of MIS in the LACC trial, along with wide adoption of robot-assisted laparoscopic surgery in high-volume tertiary oncologic centres, opened the way for a currently ongoing international multi- centre, randomized controlled trial (Robot-assisted approach to cervical cancer-RACC) (Falconer et al., 2019).

## Endometrial carcinoma

Endometrial carcinoma (EC) is the most common gynaecologic malignancy in the developed countries. Type 1 neoplasms are low-grade endometrioid ECs and are the most common type. The surgical removal of the uterus offers in early stages an excellent prognosis (Amant et al., 2005). Historically, this has been performed via laparotomy, however in the recent years there has been a clear world-wide trend towards MIS (Madhvani et al., 2019). This trend can be explained by the advantages of MIS regarding perioperative morbidity, especially in patients with higher body-mass-index (BMI), who frequently develop EC, as well as by the available data regarding the efficacy of MIS in endometrial cancer.

**Figure 1:**
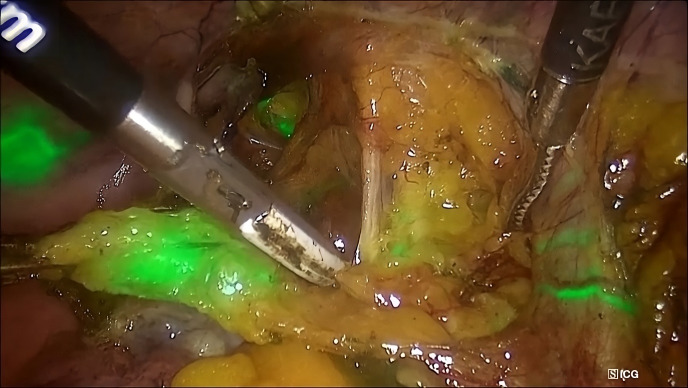
Lymphatic mapping and identification of a sentinel lymph node in the right hemipelvis of a patient with endometrial cancer utilizing the indocyanine green method and a near-infrared camera in the overlay mode.

The Gynaecologic Oncology Group LAP2 Study randomized 2616 patients, in an approximately 2:1 ratio, to a laparoscopic versus open approach for the treatment and staging of endometrial cancer (Walker et al., 2009). The main study endpoints were 6-week morbidity and mortality, hospital length of stay, conversion from laparoscopy to laparotomy, recurrence-free survival, site of recurrence, and patient-reported quality-of-life outcomes. Interestingly, twenty-five percent of the laparoscopy group were converted to laparotomy.

The most common reason for conversion was the poor visualization, but age >63, increased BMI, and presence of metastatic disease, all increased the risk for conversion. While the rate of conversion to laparotomy appears high, it is important to note that, at the time of the LAP2 trial (enrolment 1996-2005), many surgeons were relatively new to MIS for EC staging.

The median operative time for the laparotomy group was 130 minutes versus 204 minutes for the laparoscopy arm (p < 0.001). Patients in the laparoscopy group had similar rates of intraoperative complications and longer operative times (median 204 versus 130 minutes), but fewer moderate to severe postoperative adverse events (14 versus 21 percent). Fewer patients in the laparoscopy compared with laparotomy group underwent pelvic and para-aortic lymphadenectomy (92 versus 96 percent), but among those who did, there was no difference between groups in lymph node counts. There was no difference between groups in the rate of detection of advanced-stage disease (stage IIIA, IIIC, or IVB; 17 percent in both groups).

Regarding the conversion to laparotomy, subsequent patient collectives and in particular those utilizing robotic surgery, demonstrated clearly lower conversion rates (DeNardis et al., 2008; Maenpaa et al., 2016). However, this improvement can not only be attributed to the overall increase of capabilities of MIS in the recent years, but also to the use of selective or sentinel lymphadenectomy compared with protocol-directed complete lymphadenectomy in LAP2.

Later published follow-up data from the LAP2 study demonstrated that the route of hysterectomy did not affect the overall survival (five-year overall survival: 89.8 percent in both groups)(Walker et al., 2012). The five-year recurrence free survival rates were also similar (13.7 versus 11.6 percent). Importantly, quality of life (QOL) assessments and resumption of normal activities were, as expected, clearly superior in the laparoscopy group (Kornblith et al., 2009).

A later randomized trial, the Laparoscopic Approach to Cancer of the Endometrium (LACE) (Janda et al., 2017), included 760 stage I endometrioid EC patients with who underwent surgery with laparoscopic or abdominal hysterectomy. At 4.5 years of follow-up there was no difference in disease-free and overall survival. The disease-free survival rate difference was 0.3% (favouring laparoscopy; 95% CI, -5.5% to 6.1%; p = 0.007), meeting criteria for equivalence. There was no statistically significant between-group difference in recurrence of endometrial cancer (28/353; 7.9% in the abdominal hysterectomy group vs 33/407; 8.1% in the laparoscopic hysterectomy group; risk difference, 0.2% (95% CI, -3.7% to 4.0%; p = 0.93) or in the overall survival (24/353; 6.8% in the abdominal hysterectomy group vs 30/407; 7.4% in the laparoscopic hysterectomy group; risk difference, 0.6% 95% CI, -3.0% to 4.2%; p = 0.76). A subset of 361 LACE participants were enrolled in a QOL study and patients in the laparoscopic hysterectomy group reported significantly better QOL, with the improvement persisting up to six months after therapy(Janda et al., 2010).

The Cochrane Collaboration published a systematic review that included 8 studies, in which at least 70% of the patients had early-stage endometrial cancer (Galaal et al., 2012). When comparing laparoscopy to laparotomy, there was no difference in overall survival (HR 1.14, CI 0.62–2.10) or recurrence free survival (HR 1.13, CI 0.90–1.42) between the two groups. The estimated blood loss was lower in the laparoscopy group (mean difference of −106.82mL, 95% CI: −141.59, −72.06). There was also no significant difference of bladder injury (RR = 0.49, 95% CI: 0.13, 1.86), bowel injury (RR = 1.49, 95% CI: 0.39, 5.72) or vascular injury (RR = 0.43, 95% CI: 0.08 to 2.32) between laparoscopy and laparotomy. The risk of severe postoperative complications was significantly lower with laparoscopy (relative risk 0.58, 95% CI: 0.37 to 0.91).

The number of patients undergoing robotic hysterectomy for endometrial cancer is increasing (Casarin et al., 2020). A retrospective analysis of patients who underwent total hysterectomy for endometrial cancer in the United States showed that of a total of 35,224 patients, use of robotic-assisted surgery increased from 9.48% to 56.82%, while open surgery decreased from 70.4% to 28.1% between 2008 and 2015. Among propensity-score matched patients, robotic- assisted surgery was associated with shorter hospitalization than open surgery (median [interquartile range], 2.0 [2.0-3.0] vs 4.0 [3.0-6.0] days) and laparoscopic surgery (2.0 [2.0-3.0] vs 3.0 [2.0-4.0] days), fewer 30-day complications (20.1% vs 33.7%) (all p < .001). Interestingly the total 30-day perioperative cost in this study was similar: US $12,200 [US $9,509-US $16,341] for robotics vs US $12,018 [US $8,996-US $17,162]; p = 0.34) for open surgery.

DeNardis et al. (2008) compared the robotic vs. the total abdominal hysterectomy with lymphadenectomy for endometrial cancer and could demonstrate shorter length of stay, lower estimated blood loss and perioperative complication rates in the robotic cohort.

When robotic surgery for endometrial cancer was compared with the traditional laparoscopic route in a randomized controlled trial, the robotic operation was faster to perform (Maenpaa et al., 2016). The median operation time in the traditional laparoscopy group (n = 49) was 170 (range 126- 259) minutes and in the robotic surgery group (n = 50) was 139 (range 86-197) minutes, respectively (p < 0.001). In this cohort all conversions to laparotomy occurred in the traditional laparoscopy group (p = 0.027), otherwise the surgical outcome was similar between the groups and there were no differences as to the number of lymph nodes removed.

Currently there are no available data regarding the oncologic outcome of robotic vs. laparoscopic surgery, however it is fair to suggest that a non- inferiority must be expected. Additionally, the inherent capability of modern robotic systems to detect the sentinel lymph nodes, which is currently an important step of a large portion of guideline-conform hysterectomies (Concin et al., 2021), increases the practicality of this procedure (Balafoutas et al., 2020), in particular for morbidly obese patients, for which robotic surgery appears to improve the outcome in comparison to traditional laparoscopy (Chan et al., 2015).

Because of the available data, endometrial cancer is the gynaecologic malignancy with the most apparent role of MIS and therefore currently society guidelines, and in particular the guideline of the German Society of Obstetricians and Gynecologists clearly define MIS as the surgical route of choice (Emons et al., 2018).

Hysteroscopy, as part of MIS, can play an important role in fertility preservation in patients with endometrial carcinoma and desire for future childbearing. Regarding diagnosis and initial assessment, hysteroscopy guarantees the representative tumour specimen and the sufficient uterine cavity evaluation. Progestin therapy is the mainstay of fertility preservation either in the form of oral agents or in the form of a progestin-releasing intrauterine device. However, the hysteroscopic resection of early- stage EC has been described in the form of a targeted tumour removal (Giampaolino et al., 2019; Mazzon et al., 2010). Falcone et al. (2017) reported in a retrospective cohort of 28 stage IA EC patients, complete regression in 89.3% of cases and favourable reproductive outcomes with 93.3% pregnancy and 86.6% live birth rates in the 15 patients who tried to conceive. A recent review concluded that the hysteroscopic resection followed by progestins is associated with a higher complete response rate, live birth rate, and lower recurrence rate than oral progestins alone (Garzon et al., 2021).

## Ovarian cancer

A 47 –year old, otherwise healthy woman was Ovarian cancer is the second most common gynaecologic malignancy in the developed countries (Torre et al., 2015). Surgical diagnosis, staging, and treatment followed by adjuvant chemotherapy represents the cornerstone in the management of ovarian cancer. Currently, laparotomy is the standard surgical approach for the treatment of this disease.

MIS has mainly been investigated in patients with presumed stage I or II ovarian cancer in whom cytoreduction is not necessary (Tozzi et al., 2004). In a Cochrane Collaboration review, the lack of randomized controlled trials comparing laparotomy and laparoscopy for FIGO Stage I ovarian cancer was highlighted (Lawrie et al., 2013). Even for stage I tumours, capsule rupture during the procedure is a concern. However, a meta-analysis of nine studies concluded that the intraoperative rupture may not decrease progression-free survival when compared with no rupture in patients with early-stage epithelial ovarian cancer, with the limitation that these findings refer to patients who underwent complete surgical staging operation (Kim et al., 2019). In a later large observational study of 8850 patients with stage I epithelial ovarian cancer, capsule rupture occurred more frequently in patients undergoing MIS (either traditional or robotic-assisted laparoscopy) compared with laparotomy (adjusted relative risk 1.17, 95% CI 1.06-1.29) and was associated with an increased mortality in both groups (Matsuo et al., 2020). Regarding the completeness of the staging procedure, a meta-analysis found no significant differences between MIS and open surgery regarding the size of the omentectomy or the number of lymph nodes removed. Importantly, the operative time for the laparoscopy patients was significantly longer than that of the laparotomy group, however blood loss and hospital stay were shorter (Park et al., 2013). Additionally, a multicentre retrospective series from seven referral gynaecologic oncology units including 300 patients with apparent early- stage disease confirmed the advantages of MIS in terms of reduction in morbidity. In this study, the rate of recurrence, disease-free survival (DFS), and overall survival (OS) were comparable to those reported in the open surgery group (Gallotta et al., 2014). Laparoscopic and robotic surgery for ovarian cancer, compared with laparotomy, is associated with less blood loss, shorter hospital stay, and lower rates of postoperative complications (Chen et al., 2016). Robotic surgery, in particular, led to a decreased postoperative pain score. A further possible application of MIS is the evaluation and possibly the management of borderline ovarian tumours in early stages of the disease (Pecorino et al., 2023).

Regarding advanced ovarian cancer, the use of MIS for staging and not for debulking, appears to be the most researched and accepted approach (Fagotti et al., 2016). This approach might spare patients an unnecessary laparotomy resulting in suboptimal cytoreduction, with the additional advantage of obtaining biopsies for histological and possibly molecular analysis. This possibility has been investigated early on in the development of operative laparoscopy (Vergote et al., 1998), and later studies investigated the role of laparoscopy in determining whether the patients are candidates for neoadjuvant chemotherapy (Angioli et al., 2006; Fagotti et al., 2005). A Cochrane review evaluated the accuracy of the diagnostic laparoscopy in determining the resectability of disease in patients with advanced ovarian cancer (van de Vrie et al., 2019). The laparoscopy was indeed found to be a useful tool in predicting unresectable disease, but unfortunately many women were falsely predicted to have resectable disease and hence the authors concluded that it should not be considered as a standard procedure in clinical practice. A subsequent randomized controlled trial found that futile laparotomy occurred in 10 (10%) of 102 patients in the laparoscopy group versus 39 (39%) of 99 patients in the primary surgery group (relative risk, 0.25; 95% CI, 0.13 to 0.47; p < .001), suggesting that performance of diagnostic laparoscopy prior to primary debulking is reasonable (Rutten et al., 2017).

Moreover, the role of laparoscopy in interval debulking surgery has been explored recently in a retrospective cohort study using data from the National Cancer Database (Melamed et al., 2017). There was no difference in 3-year survival between patients undergoing laparoscopy [47.5%; 95% confidence interval (CI) 41.4-53.5] and laparotomy (52.6%; 95% CI 50.3-55.0; p =0.12) and the postoperative hospitalization was slightly shorter in the laparoscopy group (median 4 compared with 5 days, p<0.001), however the study could not investigate the effect of laparoscopic debulking on recurrence-free or disease-specific survival because these outcomes are not reported in the National Cancer Database. A recent retrospective propensity- matched study included 77 FIGO stage III or IV serous ovarian cancer patients, undergoing interval debulking surgery after neoadjuvant chemotherapy. No significant difference was found in terms of median OS between laparoscopy and laparotomy (23.1 months [95% CI 15.7-29.7] versus 26.3 months [95% CI 21.7-31.7], respectively, p = 0.17), suggesting that, in carefully selected patients with advanced ovarian cancer, complete laparoscopic interval debulking surgery achieves similar survival outcomes to open laparotomy (Lecointre et al., 2022). Additionally, there may be a role of MIS in the surgical management of solitary lymph node relapse in ovarian cancer cases (Pecorino et al., 2020).

In summary, the current role of MIS in ovarian cancer remains limited in specific scenarios and hence MIS in not recommended in society guidelines as the standard surgical management of this condition (Fotopoulou et al., 2017; Fotopoulou et al., 2021).

## Pelvic and paraaortic lymph node evaluation

Surgical staging of pelvic and paraaortic lymph nodes is important for the further management of endometrial, cervical, and possibly ovarian carcinoma. In cervical cancer, evaluation for pelvic and paraaortic lymph node metastases impacts prognosis and treatment decisions and belongs to staging (Cosin et al., 1998). Especially in early- stage cervical cancer, information regarding lymph node involvement helps guide whether the primary therapy will be a radical hysterectomy or a chemoradiation, so the laparoscopic lymphadenectomy can lead to the avoidance of an unnecessary median laparotomy (Gold et al., 2008; Pomel et al., 2017). The laparoscopic approach is associated with reduced morbidity (Marnitz et al., 2005; Moore et al., 2008; Sonoda et al., 2003; Zanvettor et al., 2011). In endometrial cancer, the presence of pelvic and paraaortic lymph node metastases determines whether chemotherapy with or without radiation is indicated and to what anatomical level the radiation should be administered. The possible therapeutic role of lymphadenectomy has not yet been fully clarified, however the removal of nodes harbouring metastatic disease could impact patient survival in some cases (Kilgore et al., 1995).

The surgical approach used for a lymphadenectomy procedure in gynaecologic oncology is typically determined by the best approach for the rest of the procedure, unless the lymphadenectomy will remain the sole surgical intervention. This is the case for patients with locally advanced cervical cancer (stages IB2 to IVA) who undergo pelvic and paraaortic lymphadenectomy for evaluation prior to primary chemoradiation. Technically, MIS lymphadenectomy is feasible in most cases even in the paraaortic field (Paik et al., 2020). There are two MIS methods for access to the retroperitoneum (Dargent et al., 2000). The transperitoneal approach gives better access to the pelvic nodes but renders the paraaortic lymphadenectomy more difficult in obese patients. On the contrary, the extraperitoneal approach provides excellent exposure to the paraaortic nodes, even in obese patients, but does not adequately expose the pelvic region. A systematic review of robotic extraperitoneal paraaortic lymphadenectomy included five studies and 88 patients and found that the mean number of paraaortic nodes yielded was 15.4 (standard deviation ±4.7) nodes (Bogani et al., 2016)

A sentinel lymph node (SLN) is defined as a lymph node that directly drains the primary tumour area through lymphatic channels and represents the lymph node(s) most likely to first receive lymphatic metastases. SLN detection is becoming standard in the management of the retroperitoneal lymph nodes in EC and both the NCCN (Koh et al., 2018) and the SGO (Holloway et al., 2017) support it’s utilization, even while mentioning the absence of randomized studies comparing sentinel lymphadenectomy with alternative strategies. Lymphatic mapping with blue-coloured dyes (including 1% isosulfan blue and 1% methylene blue) and technetium-99m (99mTc) have been utilized, however, due to the increasing availability of near-infrared cameras in modern laparoscopic systems and it being in the standard equipment of the last generation of robotic systems, use of indocyanine green (ICG) as a single agent has become the routine approach for detection of sentinel nodes (Zapardiel et al., 2021). The superiority of the ICG technique and its feasibility, near exclusively, with endoscopic settings further increase the role of MIS in gynaecologic cancer.

## Special issues regarding MIS in gynaecological cancer

In the field of fertility-preserving surgery for early-stage cervical cancer, the minimally invasive radical trachelectomy has emerged as an alternative to open radical hysterectomy. A recent international retrospective study concluded that the 4.5-year disease-free survival rates did not differ between open radical trachelectomy and minimally invasive radical trachelectomy (94.3%, 95% confidence interval, 91.6-97.0 and 91.5% ,95% confidence interval 87.6-95.6 log-rank p=0.37 respectively) (Salvo et al., 2022). A further retrospective international multicentre study found excellent pregnancy rates of 80% and low rates (14%) of premature delivery for patients treated with robotic radical trachelectomy (Ekdahl et al., 2022).

Port site metastasis refers to cancer growth at a port incision site after laparoscopy (Abu-Rustum et al., 2004). It is a relatively common complication of laparoscopy in the presence of intraperitoneal disseminated disease and occurs in up to 2 percent of such cases. It must be mentioned that it’s incidence is comparable with that of wound metastasis after laparotomy (Ramirez et al., 2003). Port site metastasis is a concern in particular in patients with advanced ovarian carcinoma (incidence up to 17% (Vergote et al., 2005)). It is unclear whether direct contamination by tumour cells or secondary effects from pneumoperitoneum cause this complication (Benabou et al., 2021; Ost et al., 2005) and the possible protective role of port-site excision has not been clarified (Lago et al., 2019). High-quality data on impact of port site metastasis on the prognosis are scarce, however the available data suggest lacking impact on survival (Ataseven et al., 2016).

Fader and Escobar (2009) first reported on the use of Laparo-endoscopic Single-Site Surgery (LESS) in gynaecologic oncology. Follow up retrospective series of patients report the feasibility of LESS in endometrial and ovarian cancer (Fader et al., 2010) and even in the performance of extensive lymphadenectomy (Escobar et al., 2010). A recent systematic review supports the feasibility and perioperative safety of these approaches while mentioning the lack of data regarding the long-term oncologic outcome (Russa et al., 2021). Initial data even suggest the superiority of robotic systems with single-port access for these operations (Sun et al., 2021). In our opinion the cosmetically motivated use of LESS in the treatment of cancer is exaggerated, given its limitations in endoscopic ergonomics, and should be reserved for special cases with intraabdominal pathology complicating the multi-port placement.
